# Medical Items and News of Interest to the Physician

**Published:** 1870-04

**Authors:** 


### Medical Items and News,

### Of Interest to the Profession.

CULLED FROM THE STANDARD MEDICAL JOURNALS OF THE WORLD.

NOTE.—These formulæ are intended only for the regular practitioner of medicine, and should be employed only by him, or under his immediate supervision.

**Tetanus.**

The *Bulletin de la Therapeutique* publishes two cases of recovery from tetanus by the administration of aconite.

**Chorea.**

M. Mazode, of Lyons, cures chorea by Ether-spray applied to the spine. He uses fourteen drachms of ether with Richardson's apparatus.

**Treatment of Burns.**

The Boston *Med. & Surg. Journal* says that dry earth is successfully used in the City Hospital, in the treatment of burns.

**Turkish Bath.**

The *Medical Press & Circular* records another death of a person with "heart disease," in the Turkish bath at Cork. Persons with heart ailments are warned against such baths.

**Opium Eaters.**

Dr. C. K. Ladd, of Towanda, Pa., in the transactions of the Pennsylvania Medical Society, reports the case of a man who has taken ʒ iij of opium every day.

**Iron Stoves.**

Dr. Pidtmann, in *Virchows Archives*, says that chronic poisoning by carbonic acid is very frequent in his part of the country, where iron stoves are extensively used.

**Hemicrania.**

Dr. A. H. Smith, of Philadelphia, in an article to the *Am. Jr. of Obstetrics*, speaks very highly of amon-muirat, in fifteen grain doses, repeated every two hours.

**Treatment of Ozæna.**

The *Medical Press & Circular* advises the use of permanganate of potash, five parts to one hundred of water, with the nasal douche, in the treatment of ozæna. It will remove the disgusting odor and heal the ulcerated surfaces.

**An Agreeable Remedy.**

We have seen much written recently, in the various medical journals, of the good results from the administration of water-melon in diarrhœa. Water-melon is recommended to be eaten in large quantities and will certainly cure the most severe cases of diarrhœa.

**Epilepsy.**

Geo. Johnson, M. D., F. R. C. P., and Physician to King's College Hospital, advises chloroform and bromide of potassium in the treatment of epilepsy. Chloroform he says, wards off a threatened fit, and cuts short a violent and prolonged paroxysm.

**Delirium Tremens.**

A correspondent to the Philadelphia *Medical & Surgical Reporter* extols the use of the tincture of digitalis in severe cases of delirium tremens. He advises its administration in drachm doses, every hour. The treatment of two cases are given, in this manner, both resulting in speedy cures.

**Opium Poisoning.**

We read in the *Anuales d' Electricite*, of a case of opium poisoning that resisted the employment of tannin, coffee and tartar emetic, that finally recovered from the application of the galvanic battery. The patient took 0.5 grammes of opium. The poles of the battery were placed on the neck and perineum of the patient.

**Trismus Neonatorum.**

Dr. Alois Monti of the Dr. Ann's Child's Hospital, reports in *Jarhb. fur Kinderheilk* three cases out of five cured by calabar bean. He uses the remedy by subcutaneous injection, every ten minutes until spasms cease. For a new born child he uses one tenth grain of the extract, for a dose, and gradually increases to one-third, one-half, or a whole grain a day.

**Glycerine of Tannin.**

Mr. Hill, surgeon of the London Royal Free Hospital, has furnished notes of six obstinate cases of acute and chronic gonorrhœa, to the *London Lancet*, in which glycerine of tannin was used successfully. The formula is as follows:

Glycerine of Tannin, ℥ iij;

Olive Oil,

Mucilage, $\overline{aa}$ ℥ j.

M.

**Sweet Quinine.**

Dr. Squibb pronounces this preparation a humbug—says there is no quinia in it.

---

**Sulphate of Anilin.**

A new remedy which is recommended by medical journals in chorea, hysteria, and in obstinate cases of pain in the foot. Ten grains are given three times daily.

---

**Exterpation of the Uterus.**

Prof. Langenbeck, of Hanover, has recently performed the operation upon a woman of 48. The result was good—patient removed the ligatures herself in eight days. The operation was performed on the 15th of May, and on the 29th she left her bed.

---

**Inhalation of Oxygen.**

The *Practitioner* contains a paper by Dr. E. Mackay, detailing a number of cases of phthisis and other pulmonary diseases successfully treated by inhalations of oxygen gas, diluted with common air, in proportions varying between six and twelve parts of oxygen to sixty of air.

---

**Vaginismus.**

Dr. Tilt, (*Ibid*) says there is no necessity for the use of knife or scissors in this difficulty. He advises the patient to be placed under the influence of chloroform, then separates the walls of the vagina for five minutes, with his two thumbs, placed back to back. He then introduces a large metal bougie, which is held in place with a T. bandage for a few days.

---

**Hysterical Aphonia.**

Dr. Tanner, in *Ibid*, says that he never fails to cure this obstinate nervous disease by means of electro-magnetism. The patient is required to hold one pole of the battery in her moistened hand while the Doctor touches her tongue with the other pole. The result is a scream from the patient, who at once convinces herself and friends that she has *not* lost her voice.

---

**Whooping Cough.**

The physicians of Newark, N. J., are administering Gas Works for whooping cough. Rather a large dose to take, but the prescription requires the children to spend a couple of hours daily, in the purifying room of the gas works, where the inhalations of the fumes arising from the gas passing through lime, has a tendency to shorten the disease.

**Powder for Indolent Condylomata.**

The *L'Union Medicale* contains the following formula for a dressing:

℞.

Pulv. alumin. et potassæ,
Pulv. Sabinæ, a a ℥ i.

M.

Constitutional remedies are also to be employed in conjunction with the dressing.

---

**How to give Quinia.**

A correspondent of the Philadelphia *Med. & Surg. Reporter* gives the following formula to cover the taste of quinia:

℞.

Quiniæ sulphatis, gr. xxiv;

Syrup sarsap. comp. f. ℥. iij;

Acidi tannici, gr. iij;

Ol. Menthæ Piperitæ, gtt. v.

M.

To be given in quantity to suit the case.

---

**Subcutaneus Medication in Syphilis.**

Dr. Max Van-Monshos reported in the Brussels Academy, five cases of secondary syphilis, with indurated chancres, treated by subcutaneous injection of calomel. Three of the cases received two or three injections each (quantity not stated) at intervals of about twelve days. These were nearly or quite cured after the lapse of thirty-seven, twenty, nineteen days respectively; and the two remaining cases, and which had each received one injection only eight days previously, were notably improved. If this discovery is verified, it will prove one of the most important ever made in regard to the treatment of syphilis. *Med. & Surg. Journal.*

---

**Painless Surgery.**

Dr. B. W. Richardson read a paper before the British Medical Association, on a new method of painless cutting in surgery. The author placed before the section a knife consisting of a revolving blade, and which divided with such rapidity that superficial incisions could be made with it without pain. The revolutions were about 25 per second, but the speed might be greatly increased. The knife in its action illustrated that an appreciable interval of time is necessary for fixing an impression on the mind, and for the developement of consciousness. He hoped he should soon be able to give to the surgeon a small pocket instrument with which to open abscesses, and perform many minor surgi- operations painlessly, without having recourse to either general or local anæsthesia.—*Medical Record.*

**Sedative Belladonna Plaster.**

The following will be found to be most an excellent local application in the treatment of rheumatic and neuralgic pains:

℞.
Emp. plumbi, ℥ xvj.
Ext. pini sylvestris,
Ext. belladonnæ, āā ℥ iss.
M.

Sig:—Spread evenly over strong linen, and apply warm, to the parts affected.

---

**Tteatment of Neuralgia.**

M. Bertrand, Paris, recommends the following ointment in the treatment of facial neuralgia:

℞.

Veratrine, gr. v;

Morphiæ Sulphatis, gr. iij;

Adipis, ℥ j.

M.

Sig:—Rub the painful parts with the ointment when the paroxysims of pain are at their height. Two or three frictions usually suffice.

We have found the pills of *Iodiform et ferri*, as manufactured by Wm. R. Warner & Co., wholesale chemists, at 154 North 3d. St., Phil., a most effectual remedy in the treatment of facial neuralgia. We have prescribed where all the usual remedies have failed, and have invariably succeeded in arresting the disease with the pills above named. Two pills are given ter die.

---

**Hæmorrhoids.**

Prof. Fordyce Baker is in the habit of prescribing as follows:

℞.

Sulphur sublimatum,

Magnesiæ sulphas,

Potassæ bitartras,

Magnesiæ carbonas, āā ℥ ss.

M.

Sig:—One tablespoonful before breakfast.

In stubborn cases attended with considerable constipation, the following pill is recommended:

℞.

Pulv. aloes,

Ext. nux vomica, āā gr. ss.

Fiat pil. tales. Sig:—One pill three times daily.

In hæmorrhoids complicated with slight looseness of the bowles, he advocates this prescription:

℞.
Ferri sulphas, gr. j;
Pulv. aloes, gr. ss.

Ft. pil. tales. Sig:—One pill three times daily.

**Diabetes.**

Mr. Bayfield in the *British Medical Journal*, reports a case of diabetes cured by the administration of half drachm doses of the etherial essence of the peroxide of hydrogen.

---

**Phthisic.**

A writer in the *British Medical Journal* says he has had great success in the treatment of phthisic with *cod-liver oil and ether*. He says he has several cases that are gaining rapidly under it, one having gained 7 pounds in 23 days.

---

**Arrest of Tubercle.**

Dr. Cormell of Belfast, states that the development of tubercle can be prevented by uniformly enforcing the habitual respiration of air not pre-breathed.—*Record.*

---

**Lupuline.**

M. Mehu says that the resin of the hop, in the dose of 20 grains, produces intense headache and sometimes nausea. Here then, is a remedy for our homeopathic friends, for "sick headache;" upon the principal of "*similia similibus curanter*."

---

**Milk Diet in Disease.**

The quantity of milk calculated for an adult is four pints daily. Milk is an aliment of easy and rapid digestion, which leaves little residue, favoring diseases. A milk-diet strictly followed, has rendered great service in organic affections, hypertrophy of the heart, ulcerations of the digestive tube, and dropsies.—*Cal. Med. Gazette.*

---

**To Prevent Death by Chloroform.**

Experiments on inferior animals show that they may be restored from apparent death from chloroform by the continuous galvanic current, the negative pole being put in the mouth and the positive pole in the rectum. In some cases the animal was left for two minutes in a state of apparent death and then restored.—*Record.*

---

**Malignant Pustule,**

Dr. Caspar, of Stassfurth (*Deutsche Klinik*), has administered liq. ammoniæ causticæ in several hundred cases of malignant pustule. He gives to children from one to three drops, to adults five drops, in sweetened, oatmeal gruel, every hour by day and night. He also uses dilute liq. chlorine to the ulcer. The treatment is continued until the inflammation in the neighborhood of the ulcer ceases to spread.

**Psoriasis.**

Dr. J. M. DaCosta, prescribes the following unguent, to be rubbed in morning and evening;

R.

Ung. hydrag, oxidi rubri;

Ung. hydragyri, aa ʒ ij;

Glycerinæ, f ℥ ss.

M.

**Remedy for Toothache.**

A correspondent to the *Missouri Dental Journal*, recommends the following:

R.

Ether Sulph.

Chloroform, aa qtt. xxx.

Tinct. Camph. ʒ i;

Tinct. Opii, ʒ ij.

Alcohol, ℥ ss.

M.

Sig:—Apply on cotton.

**Delirium Tremens.**

Dr. Denton, (in the *Practitioner*), records two cases of well marked delirium tremens which were successfully treated by five to ten minim doses of tincture of nux vomica, repeated every four hours, after having first used an active cholagogue purgative.

**Local Treatment of Croup.**

Dr. Weber, of Darmstadt, has recently been employing *lactic acid* in croup, to the use of which he was led by noticing its remarkable power of disolving fibronous exudations. In the first instance he adopted it to clear away the croupal membranes which collected in the canula after the performance of tracheotomy. The beneficial results were so great that he proceeded to apply it locally before attempting to operate, and although he receives many cases of this disease into his wards he has never had any occasion since commencing its use to open the trachea. The mode of application of the lactic acid is by means of the inhalation apparatus, from 10 to 20 drops in half an ounce of water being inhaled, at first every half hour, and then in proportion as the breathing is releaved, the quantity being reduced from ten to five drops in the same quantity of water. He never found it requisite to continue the remedy for more then twelve hours. Usually after the application has been repeated for a few times, he discontinued it, and replaced it by camomile tea. The eyes and other parts of the face must be carefully protected from the steam, or its cauterizing effects becomes immediately apparent.—*Lancet.*

